# Glimpse: A Gaze-Based Measure of Temporal Salience

**DOI:** 10.3390/s21093099

**Published:** 2021-04-29

**Authors:** V. Javier Traver, Judith Zorío, Luis A. Leiva

**Affiliations:** 1 Institute of New Imaging Technologies, Universitat Jaume I, Av. Vicent Sos Baynat, s/n, E12071 Castellón, Spain; 2 Universitat Jaume I, Av. Vicent Sos Baynat, s/n, E12071 Castellón, Spain; al258412@alumail.uji.es; 3 Department of Computer Science, University of Luxembourg, Belval, 6 Avenue de la Fonte, L-4264 Esch-sur-Alzette, Luxembourg; luis.leiva@uni.lu

**Keywords:** visual attention, temporal salience, salience maps, eye-gaze, video

## Abstract

Temporal salience considers how visual attention varies over time. Although visual salience has been widely studied from a spatial perspective, its temporal dimension has been mostly ignored, despite arguably being of utmost importance to understand the temporal evolution of attention on dynamic contents. To address this gap, we proposed Glimpse, a novel measure to compute temporal salience based on the observer-spatio-temporal consistency of raw gaze data. The measure is conceptually simple, training free, and provides a semantically meaningful quantification of visual attention over time. As an extension, we explored scoring algorithms to estimate temporal salience from spatial salience maps predicted with existing computational models. However, these approaches generally fall short when compared with our proposed gaze-based measure. Glimpse could serve as the basis for several downstream tasks such as segmentation or summarization of videos. Glimpse’s software and data are publicly available.

## 1. Introduction

Visual salience (or saliency) refers to the ability of an object, or part of a scene, to attract our visual attention. The biological basis for this phenomenon is well known [1]: salience emerges in parallel processing of retinal input at lower levels in the visual cortex [2]. Concepts other than salience, such as surprise [3], have been found to explain human gaze in dynamic natural scenes. While the concept of *spatial* salience has been extensively investigated for static contents such as natural images [4,5] and graphic displays [6,7], the *temporal* salience of dynamic contents such as videos remains largely unexplored. Spatial salience predicts *where* attention is allocated in the image domain, whereas temporal salience predicts *when* attention happens and *how* it varies over time.

The importance of temporal salience to gain valuable insights about a video structure has been recently noted, and a mouse click-based interaction model was proposed to annotate datasets in the absence of an eye tracker [8]. However, the approach requires significant manual work and recommends several passes over the same video to ensure a low intra-observer variability and obtain more reliable estimates.

In this work, we investigated how to automatically estimate temporal salience in videos using eye-tracking data. Our main hypothesis was that when gaze coordinates are spatio-temporally consistent across multiple observers, it is a strong indication of visual attention being allocated at a particular location within a frame (spatial consistency) and at a particular time span (temporal consistency). In other words, uninteresting dynamic contents are expected to induce non-homogeneous, randomly located gaze points (low temporal salience), whereas truly attention-grabbing contents would concentrate similar gaze points from different observers for some time span (high temporal salience).

**Figure 1:**
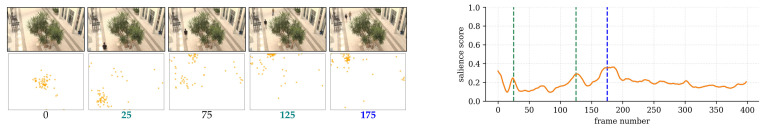
Demonstrating Glimpse with video v22 of the SAVAM dataset. (**Left**): From top to bottom: source video frames, observers’ gaze points, and frame numbers. (**Right**): temporal salience score estimation with pointers to some key events.

Our approach, named Glimpse (gaze’s spatio-temporal consistency from multiple observers), is illustrated in Figure 1. As can be observed, there is a general agreement between the estimated low-level temporal salience and the high-level visually salient events in the video. At the beginning, a center bias is identified (this happens in most SAVAM videos, as a result of the experimental conditions; see https://compression.ru/video/savam/ last accessed on 28 April 2021).

Then, at t≈25 and t≈125, two persons enter the scene, respectively, which correlates with the corresponding salience peaks (marked in green). The maximum salience occurs around frame t≈175 (marked in blue), where the two persons get closer and greet each other. After that, a group of people enters the scene, which draws the attention of fewer observers, and so, salience decreases accordingly.

To the best of our knowledge, Glimpse is the first method that addresses the problem of computing temporal salience from gaze data, without requiring explicit human annotation effort, nor model training. Additionally, because eye-tracking data are not always available, a secondary research contribution we made in this paper was exploring whether frame-level spatial salience maps, as predicted by existing computational models, can be used to produce reasonable estimates of temporal salience according to our method. The idea is similar as before: spatio-temporal consistency in the spatial salience map across time might provide cues for estimating temporal salience. This alternative is highly relevant because, if proven effective, it would pave the way for a more agile computation of temporal salience, without having to recruit human participants.

In sum, the key contributions of this paper are: (1) a measure of temporal salience in dynamic scenes based on the notion of observer-spatio-temporal gaze consistency; (2) analysis and evaluation of the proposed measure; (3) an exploration of heuristic measures of temporal salience derived from computational models of spatial salience; and (4) software and data to allow others to build upon our work.

## 2. Related Work

Our work was mostly related to research on eye-tracking applications in dynamic scenes, such as segmentation, summarization, and compression of videos. We review those here and also relate to recent tools that have been used for annotation of temporal salience datasets.

### 2.1. Downstream Applications

Many video summarization approaches rely on predicting frame-level importance scores [9,10], which are task dependent and therefore biased towards a particular summarization goal, whereas temporal salience is a more generic concept that could in turn be tailored to more specific or higher level tasks. Since eye gaze is known to provide cues on the underlying cognitive processes [11,12], it can be expected to be particularly useful for this kind of video-processing task. However, despite being used in some computer vision problems [13,14,15,16], its general use has been limited.

Gaze data in first-person wearable systems can aid in temporal video segmentation [17], and its computational prediction has been studied [18]. The gaze data of the wearer of an egocentric camera have been used to score the importance of the frames, as the input to a fast-forward algorithm [19]. In these cases, however, gaze is available only from a single user [17,19] (the wearer), instead of the (multiple) watchers of a video, as considered in our work.

An alternative to estimating the intrinsic salience of the visual contents is to analyze the observers’ attention, as in a recent work [20], which found that the eye movements of students watching instructional videos were similar. It has also been found that gaze location may vary upon repeated viewings of the same video [21]. In the scope of behavioral biometrics, the fusion of mouse and eye data has been proposed for improved user identification [22].

### 2.2. Handling Temporal Information

Low-level conspicuity maps can be used to derive a temporal attention curve [23], to subsequently extract keyframes through a clustering-based temporal segmentation based on the visual similarity of neighboring frames. A similar approach has been proposed [24], but including camera motion as a visual feature, plus audio and linguistic cues. These approaches are arguably difficult to use in real-time applications.

Salience maps derived from gaze data can be used for video compression by preserving higher visual quality at salient spatial regions. Based on the notion of the temporal consistency of attention (i.e., spatial salient regions in neighboring frames are likely to overlap), a temporal propagation of the salience map can be performed [25]. Although the temporal concept is indirectly considered, salience is used as a purely spatial concept.

The recently introduced concept of multi-duration salience [26] includes a notion of time, but still for defining spatial maps for static contents. Salience in dynamic scenes is related to but conceptually different from salience in static images [27]. Specific methods for the dynamic case have been studied [28,29,30,31,32,33] and, very recently, unified image-video approaches [34] proposed, but only in the context of spatial salience. For gaze prediction, temporal features are found to be of key importance in rare events, so spatial static features can explain gaze in most cases [35]. At the same time, features derived from deep learning models exploiting temporal information have been found to benefit gaze estimation over using static-only features [36].

### 2.3. Annotation Tools

Finally, researchers have sought different annotation approaches for understanding and predicting visual attention, mostly focused on static images [37]. Crowdsourcing techniques such as the Restricted Focus Viewer [38] or BubbleView [39] have emerged as a poor man’s eye tracker [40] to collect data at a large scale, where the computer display is blurred and the user has to move or click their mouse in order to see a small region in focus [8,41]. User-unknown limited mouse clicks [8] require the same participant to watch the same contents several times. This brings more reliable annotation, but challenges its scalability in terms of the length or number of videos. A comprehensive review of user interfaces for predicting stimulus-driven attentional selection [42] and a comparison of recent methodologies [43] are representative of alternatives to eye-based data.

### 2.4. Novelty and Relevance of Glimpse

It is important to highlight the novelty of both the problem addressed in this work (*temporal* quantification of the visual salience of *dynamic* contents) and the proposed approach (measure based on observer-spatio-temporal consistency of gaze data) with respect to these previous works. Specifically, some existing approaches consider time-varying contents (videos), but only estimate spatial salience maps, without providing a scalar salience score as a function of time. Furthermore, when the temporal dimension is considered, it is only for the purpose of improving the quality of the estimated spatial salience maps. Even the recent concept of multi-duration salience, it is still based on the notion of spatial maps and for static contents. Additionally, based on gaze data, Glimpse is fundamentally different from explicit human-annotation-based approaches.

Because of all these reasons, Glimpse is the first of its kind, to the best of our knowledge. As happens with new problems and approaches, this novelty prevents us from quantitatively assessing its performance (no prior ground-truth exists yet), but it also represents a unique opportunity to provide the scientific community with a reference quantification of temporal salience in terms of both ready-to-use measures computed on a particular video dataset and software to measure temporal salience for other dynamic contents with available gaze data. We believe this will significantly facilitate research progress on the problem of temporal visual salience estimation and its applications.

## 3. Measure Description

We illustrate Glimpse with videos, the paradigmatic example of time-varying visual contents. For a given video, let g(o,t)=(x,y) be the gaze position of observer o∈{1,…,N} at time (or frame number) t∈{1,…,T}, for *N* observers along the *T* frame long video. There are four variables involved: two spatial coordinates (x,y), the temporal domain *t*, and the observer *o*. Our goal is to compute a temporal salience score s(t)∈R for each frame *t* from the (implicit) four-dimensional function f(x,y,o,t).

The idea is to capture the spatio-temporal consistency of the observers’ gaze points. This entails some notion of the distance and dispersion of such points distributions: the closer they are, both in space and time, the higher the consistency. After some exploration, we were eventually inspired by Ripley’s *K* function [44], a measure of spatial homogeneity that has been used, for example, in ecology [45] and bio-geography [46]. Based on this measure, we formulated temporal salience as:

(1) $$s\left(t\right)=\frac{2}{n(n-1)}\sum_{\begin{array}{c} i,j\in \{1,\ldots ,n\}\\ i\neq j \end{array}}𝟙\left[d_{ij}<\theta_{s}\right],\;t\in \left\{1,\ldots ,T\right\},$$

where $d_{ij}$ is the pairwise Euclidean distance between the *i*th and *j*th points in the set $P_{t}$ of *n* gaze points from all the observers within a temporal window of length $2\theta_{t}+1$ centered at *t*, i.e.,

(2) $$P_{t}=\left\{g\left(o,t\right):o\in \left\{1,\ldots ,N\right\},\;t\in \left[t-\theta_{t},t+\theta_{t}\right]\right\},$$

and 𝟙[p] is the indicator function, which is one when predicate *p* is true and zero otherwise. We used $\theta_{s}$ to denote the spatial scale, which is a distance threshold. Thus, Equation (1) accounts for the number of paired gaze points that are close enough, in a normalized way, so that s(t)∈[0,1]. The larger s(t) is, the higher the spatio-temporal and inter-observer consistency, which in our problem translates to higher temporal salience.

This definition of s(t) is interesting because, besides being rather natural and relatively simple, it implicitly captures an aggregation measure without the need for an explicit clustering, which would be more computationally expensive as well. Note that in this definition of s(t), there is no need to keep track of which gaze points belong to which observer: all gaze points within the specified temporal window can be considered as a “bag of gaze points”. Furthermore, importantly, the gaze points are processed in raw form, i.e., without computing gaze features, nor classifying gaze points into fixations or saccades.

## 4. Evaluation

We tested Glimpse with the publicly available SAVAM dataset [25]. Details on the eye tracker, videos, and users watching those videos are given in Table 1. Since we were interested in frame-level data and the frame rate of the videos was smaller than the eye tracker’s sampling frequency, gaze positions within a frame were averaged. The data were recorded using a binocular system, so we arbitrarily chose the left eye as the input source.

The (x,y) gaze coordinates were normalized to [0,1] by dividing them by the frame’s width *W* and height *H*, respectively. This makes Glimpse independent of the video frame size and facilitates setting a meaningful distance threshold $\theta_{s}$ across studies.

### 4.1. Analysis of Hyperparameters

**Figure 2:**
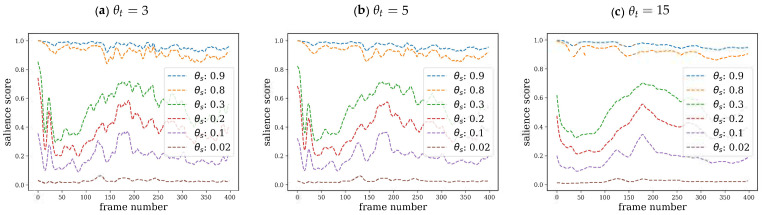
Effect of spatial scale $\theta_{s}$ and temporal scale $\theta_{t}$ on salience score s(t) computed with Glimpse for SAVAM video v22 (see Figure 1 for an example of the video contents).

We first studied the effect of the spatial $\theta_{s}$ (distance threshold) and temporal $\theta_{t}$ (time window) parameters of Glimpse. As shown in Figure 2, for a fixed $\theta_{t}$, a too permissive or a too strict distance threshold $\theta_{s}$ leads to salience estimates that are either nearly always too low or too high, which are essentially uninformative.

For intermediate values of $\theta_{s}$, the score profile s(t) is similar, but larger values of $\theta_{s}$ produce generally higher salience scores. In addition, some particular values induce better discrimination between peaks and valleys. Regarding the effect of the temporal window $\theta_{t}$ for the same spatial scale $\theta_{s}$, an increase in $\theta_{t}$ produces a smoothing effect on salience estimates. We empirically set $\theta_{s}=0.1$ and $\theta_{t}=5$ as reasonable values, according to earlier pilot experiments.

### 4.2. Convergence Analysis

Now, for fixed values of the hyperparameters ($\theta_{s}=0.1$, $\theta_{t}=5$), we conducted a convergence analysis to see how many observers would be required to get salience profiles as close as possible to those obtained with all the observers, which would be the best case scenario but also the most expensive overall, as it requires more human participants.

Let $s_{k}\left(t\right)$ be the salience scores produced for 1≤k≤N observers. We wanted to compare $s_{k}\left(t\right)$ to $s_{N}\left(t\right)$ for a given video of length *T*. To this end, the length-normalized Euclidean distance *d* between two salience scores, $s_{1}\left(t\right)$ and $s_{2}\left(t\right)$, is defined as:

(3) $$d\left(s_{1},s_{2}\right)=\sqrt{\frac{1}{T}\sum_{t=1}^{T}{\left[s_{1}\left(t\right)-s_{2}\left(t\right)\right]}^{2}}.$$

We took p(k)=min(pmax,Nk) random samples of size *k* observers out of the *N* observers available in the SAVAM dataset. A conservative upper bound of $p_{\max}=400$ was set so that not all possible combinations were computed (for example, for N=58 observers, there are as many as 30,856 different combinations of k=3 observers) and computed the mean of $d_{k,N}=d\left(s_{k},s_{N}\right)$ for each of the samples.

**Figure 3:**
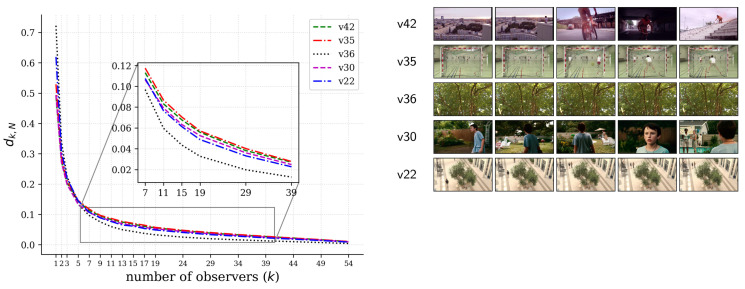
Convergence analysis for assessing the scalability of Glimpse in terms of the number of observers. These curves correspond to five different videos, and the convergence trend is similar across the SAVAM videos.

**Figure 4:**
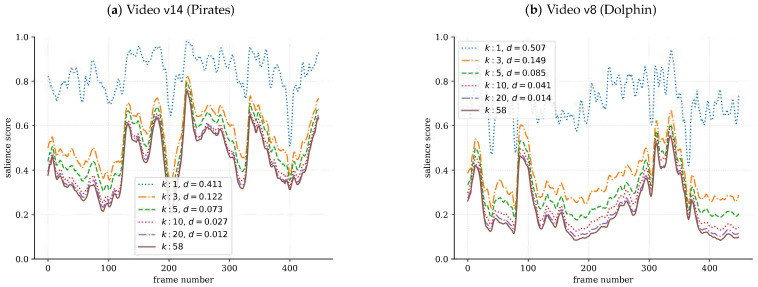
Temporal salience score $s_{k}$ with Glimpse for a varying number of observers *k*, together with their corresponding distance to $s_{N}$, $d_{k,N}$, when considering all observers. Examples for videos (**a**) v14 and (**b**) v8.

**Figure 5:**
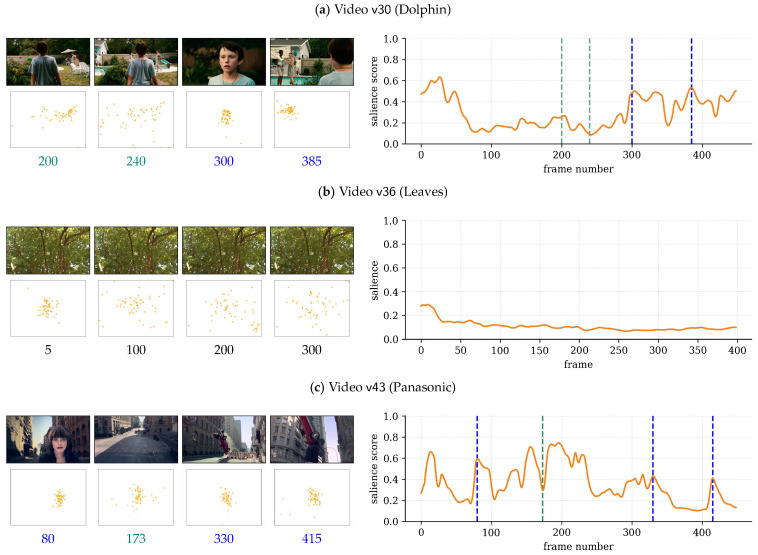
Examples of temporal salience scores s(t) on some SAVAM videos: (**a**) v30, (**b**) v36, and (**c**) v43.

As shown in Figure 3, convergence happens quickly, which means that a reliable temporal salience can be obtained with much fewer observers, suggesting thus that Glimpse is quite scalable. The confidence intervals are very small and hence not shown in the figure. A very similar trend was observed for the rest of the SAVAM videos. As a reference for the scale of the distance, it is worth looking at Figure 4 (discussed below), which shows the related profiles of signals $s_{k}\left(t\right)$ and $s_{N}\left(t\right)$, together with the corresponding distances $d_{k,N}$. It can be noted in Figure 3 that $d_{k,N}$ is particularly low for k>5 for video v36, which has a (almost constant) low salience score along the whole video; see Figure 5b. This result is particularly relevant since it can be expected that for low-attention contents, less observers are required to get reliable estimates of temporal salience.

### 4.3. Effect of the Number of Observers

Now, we use Figure 4 to illustrate temporal salience scores $s_{k}\left(t\right)$ for a varying number of observers *k*. Two main observations are worth mentioning: First, it can be seen that $s_{k}\left(t\right)\geq s_{k^{\prime }}\left(t\right)$ for $k<k^{\prime }$; i.e., the fewer the observers, the more overestimated the salience score tends to be, and therefore, $s_{N}\left(t\right)$ represents a conservative lower bound. Second, the convergence of $s_{k}\left(t\right)$ to $s_{N}\left(t\right)$ with *k* is quite apparent, reinforcing the fact that it happens with very few observers, as noticed before in Figure 3.

### 4.4. Qualitative Assessment

Similar to Figure 1, which illustrates Glimpse for video v22 in the SAVAM dataset, further examples are provided in Figure 5 that highlight the behavior of the proposed measure.

In v30, at t≈200 the woman in the background grabs the attention of many observers, with the corresponding increase in the salience score; see Figure 5a. Afterwards, at t≈240, attention spreads across the woman, the back of the boy, and other scene regions, resulting in lower salience scores. Then, at t≈300 and t≈380, attention is highly consistent around the boy’s face and at the women in the background, respectively, and so, s(t) exhibits local peaks at those times.

In v36, the tree leaves are moving with the wind all the time, with no particular region drawing the observers’ attention; see Figure 5b. Consequently, gaze locations are not homogeneous, and accordingly, the salience score is very low and flat overall.

The more dynamic contents in v43 produce higher peaks and more variations in the temporal salience than in other examples; see Figure 1 and Figure 5. The high score at t≈80 aligns with the appearance of the girl’s face. The valley at t≈173 can be explained by a scene change, where observers’ gaze points diverge. An eye-catching car maneuver draws the attention of the observers around t≈330 and, after a viewpoint change, again at t≈415.

The qualitative results reported in [8] (Figure 3) include the salience scores for 250 frames of videos v30 and v36 (Figure 5). Like our approach, their salience scores in video v30 are higher in accordance to relevant video events. However, Glimpse differs in *when* peaks and valleys happen in the salience signal, as well as the overall salience scores, in absolute terms. For v36, their scores are essentially flat, as with Glimpse. However, their scores are close to 0.5 in some parts, whereas Glimpse predicts much lower scores overall (about 0.1), which arguably reflects better the “monotonous” content of this video.

### 4.5. Comparison with Downstream Applications

Since currently there is no ground-truth or alternative approaches for temporal salience computation, a fair quantitative comparison is not possible. However, as a reference, we compared Glimpse with two approaches of video-related problems, namely a popular temporal segmentation approach, Kernel-based temporal segmentation (KTS) [47], and a recent memory-based frame-wise visual interestingness estimation method [48] (VisInt). The input to KTS were the 2048-dimensional activations prior to the last fully connected layer of InceptionV3 [49] from the video frames downsampled to a 400×225 resolution. The input to VisInt was the video frames resized to 320×320 resolution, as per the default choice in the authors’ software (https://github.com/wang-chen/interestingness last accessed on 28 April 2021).

The salience score s(t) from Glimpse was compared with a reference signal r(t). For KTS, r(t)=1 for frames temporally close to the detected scene change points, and r(t)=0 otherwise. For VisInt, r(t) is the interestingness score. We tested several of VisInt’s writing rates ($\gamma_{w}>0$) to the visual memory system during online learning, where higher $\gamma_{w}$ implies decreasing interest in new visual inputs earlier.

On the one hand, we compared Glimpse with KTS using the precision and recall metrics, since the KTS signal is binary, defined as precision=I/S and recall=I/R, with $I=\sum_{t}\min\left(s\left(t\right),r\left(t\right)\right)$, $R=\sum_{t}r\left(t\right)$, and $S=\sum_{t}s\left(t\right)$. Both metrics are defined in [0,1], with lower values representing a poor match between the compared signals. On the other hand, we compared Glimpse with VisInt using Spearman’s ρ and Kendall’s τ, since both are continuous signals, and they were recommended in similar contexts [50]. Both rank correlation metrics are defined in [−1,1], with values close to zero denoting weak or no correlation.

**Figure 6:**
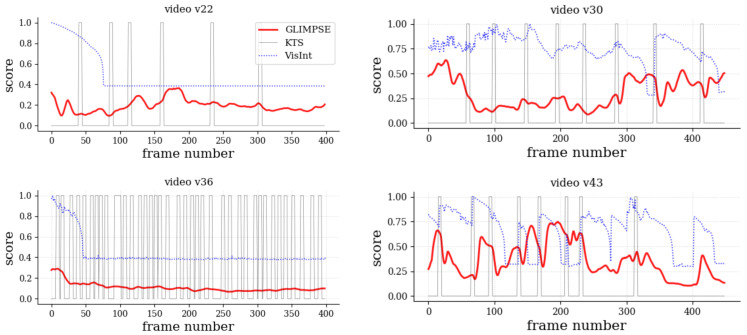
Outputs of Glimpse, KTS, and VisInt (normalized to [0,1] and using $\gamma_{w}$ = 0.2).

Results across SAVAM videos revealed low precision and recall, as shown in Table 2a, and essentially no correlation; see Table 2b. This means that Glimpse differs from other segmentation- or “importance”-like scoring approaches. In particular, it can be observed in Figure 6 that interestingness peaks from VisInt tend to agree (v30, v43) with some scene change points detected by KTS, but Glimpse is not biased by these changes. On the one hand, VisInt produced a flat signal in v36, which rightfully corresponded to the homogeneous contents of that video, but it also did so in v22, thus missing the subtle image changes corresponding to people moving in the hall (Figure 1) and that Glimpse aptly captured. On the other hand, KTS may produce non-meaningful scene changes (v22, v36) and did not align with attention-grabbing moments, as detected by Glimpse (v30 and v43).

In sum, these experiments highlighted how existing scoring techniques for detecting key events rely on low-level visual cues and tend to produce suboptimal results at best. In contrast, being based on the cognitively rich human gaze, Glimpse was able to robustly estimate the temporal evolution of attention in a semantically meaningful way.

In terms of computational efforts, asymptotic costs (Table 3) indicated that Glimpse and VisInt, being online algorithms, depend linearly on the length of the video *T*, whereas KTS has a quadratic dependency and might scale poorly to long videos. The cost for KTS did not include the part of extracting the frame features. Glimpse had a quadratic term for the number of gaze points *n* within a temporal window, which can be in the order of a few hundred (e.g., for N=58 observers and $\theta_{t}=5$ frames in our experiments). Since gaze points are very low-dimensional (simply 2D), computing the pair-wise distances is very efficient. Once gaze points were available, Glimpse was really fast, since it did not depend on either the size of the frames or the video length, unlike VisInt, which had video frames as the input, or KTS, which usually deals with long frame feature vectors. Actual running times (Table 4) highlighted how efficient Glimpse was: about one order of magnitude faster than KTS (even without feature extraction) and more than two orders of magnitude faster than VisInt. These statistics corresponded to times measured for the first 10 videos (v01–v10) in the SAVAM dataset (avg. number of frames per video: 444.0±15.8), using an AMD Ryzen 5 processor (3550H series) @ 2.1 GHz with 8 GB of RAM and a built-in NVIDIA GeForce GTX 1650 GPU with 4 GB of memory.

### 4.6. Summary

Glimpse provides a consistent quantification of temporal salience, with good convergence behavior in terms of the number of observers required to achieve temporal scores similar to those of many more observers. This is particularly interesting, since with Glimpse, it is not necessary to recruit many users who can provide eye-tracking data: with as few as three observers, we can expect an average error as small as 1%. Additionally, our qualitative experiments showed that Glimpse produced temporal salience estimates that were well aligned with key attention-grabbing events in the videos, unlike other downstream video applications (temporal segmentation, interestingness estimation), which have different purposes. This also suggested that this kind of gaze-based measure cannot be easily replaced by existing low-level algorithms relying only on purely visual cues. We concluded that Glimpse contributes to understanding how salience evolves in dynamic scenes, which can enable or assist several downstream applications such as the ones discussed in Section 2.

## 5. Experiments with Computational Salience Models

Since Glimpse provides a consistent and reliable reference of temporal salience, we investigated whether temporal salience can be alternatively estimated from spatial salience maps predicted by computational models. In the literature, these models have been shown to correlate reasonably well with human fixations [51], but it is still unknown whether they can be used to derive reliable temporal salience scores. We explored this possibility by considering several existing computational models of spatial salience (Section 5.1); some heuristic scoring algorithms (Section 5.2) that map spatial salience in the 2D image domain to 1D salience scores in the time domain; and then comparing their output (Section 5.3) when Glimpse is taken as a (ground-truth) reference.

### 5.1. Models

We considered three computational models of spatial salience, each representing a family of approaches. Classic computational models such as Itti et al. [4] approached human visual attention by heuristically defining conspicuity maps that rely on locally distinctive features (e.g., color, intensity, etc.), whose combination results in a bottom-up salience map. Graph-based visual salience (GBVS) [52] is a popular model that was reported to outperform classic methods and has been tested for combining salience maps and eye fixations for visualization purposes [53]. Therefore, GBVS was the first model we selected.

Recently, deep convolutional neural nets have been proposed to predict salience maps as their output [54]. Alternatively, “salience maps” of the deepest layers in neural networks are explored not for attention modeling, but mainly for visualization and explanatory purposes [55,56]. We tested two of such deep learning models: the multiduration model [26], which predicts how the duration of each observation affects salience, and the temporally-aggregating spatial encoder-decoder network (TASED) [32], which was proposed as a video-specific salience model.

We note that the multiduration model [26] makes predictions for horizons of 0.5, 3, and 5 s. Since we observed that the resulting salience maps were not very different for our purposes, we used the 3 s horizon, which corresponds to the intermediate value. In all cases, we refer to S(x,y;t) as the spatial salience at position (x,y) and at frame *t*. Notice that this notation for the 2D spatial map *S* is different from s(t), which we use to refer to the 1D temporal salience.

### 5.2. Scoring Algorithms

**Figure 7:**
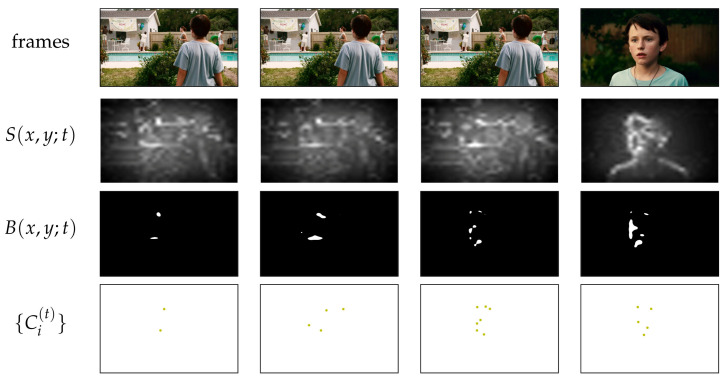
The Points scoring algorithm works by hallucinating “gaze hypotheses” points from salience maps, in this case computed by GBVS for the SAVAM v30 (dolphin).

The goal of the scoring algorithms proposed here is to produce a temporal salience score s(t) from the spatial salience maps S(x,y;t). We observed that some computational models tended to produce very noisy salience maps, while others estimated very clean salience maps. We also remark that the data variability that arises naturally with gaze points from multiple observers was lacking most of the time in the computed salience maps. These issues can be (partially) addressed differently via the following strategies:

**MutualInfo**  Comparing neighboring salience maps. The similarity of salience maps that are close in time should be able to capture the temporal consistency even when the spatial salience is noisy or spread out. This can be quantified by the (average) mutual information *I* computed over a temporal window:
  (4) $$s\left(t\right)=\frac{1}{2\theta_{t}}\sum_{k=-\theta_{t}}^{\theta_{t}}I\left(S(x,y;t),S(x,y;t+k)\right),$$
  where $\theta_{t}=5$ in our experiments, as discussed in Section 3.
**MaxValue**  Using the maximum spatial salience score. When the salience map is clean and does not vary substantially over time, the spatio-temporal consistency can be unusually high. Therefore, instead, its global maximum can be a rough indication of how salient the corresponding frame is:
  (5) $$s\left(t\right)=\max_{x,y}S\left(x,y;t\right).$$
**Spread**  Quantifying the spread of the salience map. The spatial distribution of a salience map S(x,y) is a measure of spatial consistency. To quantify this, the salience centroid $\left(x_{c},y_{c}\right)$ is first computed through weighted averages for each spatial coordinate:
  (6) $$\left(x_{c}^{\left(t\right)},y_{c}^{\left(t\right)}\right)=\left(\frac{\sum_{x,y}x\cdot S\left(x,y;t\right)}{\sum_{x,y}S\left(x,y;t\right)},\frac{\sum_{x,y}y\cdot S\left(x,y;t\right)}{\sum_{x,y}S\left(x,y;t\right)}\right),$$
  and then, the salience map is weighted with a 2D Gaussian kernel $G_{\sigma }\left(x,y\right)$ centered at $\left(x_{c},y_{c}\right)$:
  (7) $$s\left(t\right)=\frac{\sum_{x,y}S\left(x,y;t\right)\cdot G_{\sigma }\left(x-x_{c}^{\left(t\right)},y-y_{c}^{\left(t\right)}\right)}{\sum_{x,y}S\left(x,y;t\right)}.$$
  The Gaussian’s bandwidth σ dictates how tolerant it is to spread deviations (the lower σ, the more strict), similar to the role that $\theta_{s}$ has in Equation (1). We set $\sigma =W\frac{\theta_{s}}{2}$ as a function of the salience map size (width *W*) and the side length *ℓ* of the Gaussian window as ℓ=2⌈2σ⌉+1, following official implementations in computer vision toolboxes (see, e.g., https://mathworks.com/help/images/ref/imgaussfilt.html last accessed on 27 April 2021).
**Points**  Generating point hypotheses. The fact that some salience maps are noisy can be leveraged as a way to generate multiple point hypotheses and thus naturally induce some variability in the data, somehow mimicking what happens when dealing with actual gaze points from several observers. The procedure is illustrated in Figure 7 and summarized as follows:
  1. The salience map *S* was thresholded to get a binarized map *B*.
  2. The centroids $\left\{C_{i}\right\}$ of the regions (connected components) of the binary salience map *B* were computed.
  3. The Ripley-based measure (Equation (1)) was used as is, simply by replacing the gaze points in Equation (2) by these centroids $\left\{C_{i}\right\}$, also over a temporal window $\theta_{t}$.

### 5.3. Results

We compared the results of different combinations of computational model and scoring algorithm to produce estimates of temporal salience. We use the Model/Scoring notation to denote each combination. For example, GBVS/MutualInfo indicates that the spatial salience maps produced by the GBVS computational model were compared with the mutual information as the scoring algorithm.

#### 5.3.1. Quantitative Assessment

We compared the salience scores computed by a salience map model $s_{\mathrm{map}}$ against the reference salience scores $s_{\mathrm{gaze}}$ computed by Glimpse with gaze points, using $\theta_{s}=0.1$ and $\theta_{t}=5$, as above. We computed the average Jaccard index, also known as the intersection over union (IoU):

(8) $$\mathrm{IoU}\left(s_{\mathrm{map}},s_{\mathrm{gaze}}\right)=\frac{1}{T}\sum_{t=1}^{T}\frac{\min\{s_{\mathrm{map}}\left(t\right),s_{\mathrm{gaze}}\left(t\right)\}}{\max\{s_{\mathrm{map}}\left(t\right),s_{\mathrm{gaze}}\left(t\right)\}},$$

which is defined in [0,1] and has meaningful semantics [57]. IoU is widely used in computer vision for various tasks such as object detection [58]. Since different metrics capture different aspects of the compared signals, we also computed Spearman’s ρ,∈[−1,1], which accounts for non-linear correlations [59]. We observed very similar results with other similarity and correlation measures; therefore, we only report IoU and ρ for brevity’s sake. Finally, we included s(t)=λ as a straightforward baseline method, where λ∈[0,1] is a constant score of temporal salience. Note that, being constant, correlation measures cannot be computed for these baselines.

**Figure 8:**
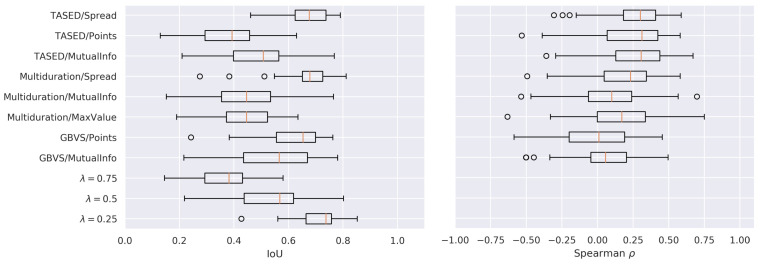
Performance results for different computational models, comparing $s_{\mathrm{map}}\left(t\right)$ with $s_{\mathrm{gaze}}\left(t\right)$.

It can be observed in Figure 8 that, overall, the performance of the computational models was rather modest. However, taking into account their limitations, in some cases, these models produced reasonable estimates. For example, for some videos and some algorithms, the IoU was as high as 0.8. As expected, there was no single best combination of a computational model and a scoring algorithm. Rather, some combinations outperformed others in some cases.

Focusing on the scoring algorithms, MutualInfo tended to perform sub-optimally when compared to most of the other combinations. Spread, in combination with the salience maps produced by both deep learning models (TASED and multiduration), achieved the highest performance. Interestingly, the Points scoring algorithm in combination with the otherwise noisy salience maps obtained with GBVS provided a very effective procedure: GBVS/Points closely followed multiduration/Spread and TASED/Spread.

The baseline method with λ=0.25 achieved the highest performance in terms of IoU, and only the three best performing algorithms outperform the baseline method with λ=0.5. There are two important aspects that constitute a good s(t) signal: one is the absolute values, which should be close to the expected temporal salience score; the other is the relative changes, which should capture when (and how much) the temporal salience increases and decreases. The simple baseline, with a properly guessed λ, might be good in the first aspect, but ignores completely the second aspect. Since the IoU metric focuses more on the absolute aspect, a better way of capturing the relative aspect would be necessary in order to compare different approaches. Regarding Spearman’s ρ, all methods had a positive, but low correlation, with those using TASED salience maps performing slightly better.

These experiments suggested that, by considering the temporal signals $s_{\mathrm{map}}\left(t\right)$ globally, the computational models behaved poorly and hardly matched $s_{\mathrm{gaze}}\left(t\right)$. As our qualitative analysis below illustrates, the temporal salience scores derived from computational models aligned relatively well with gaze-based scores only locally, i.e., at some temporal segments at some videos. As a result, these isolated locally good performances were eventually dismissed with the (globally-averaging) metrics such as *r*, ρ, and IoU.

#### 5.3.2. Qualitative Assessment

**Figure 9:**
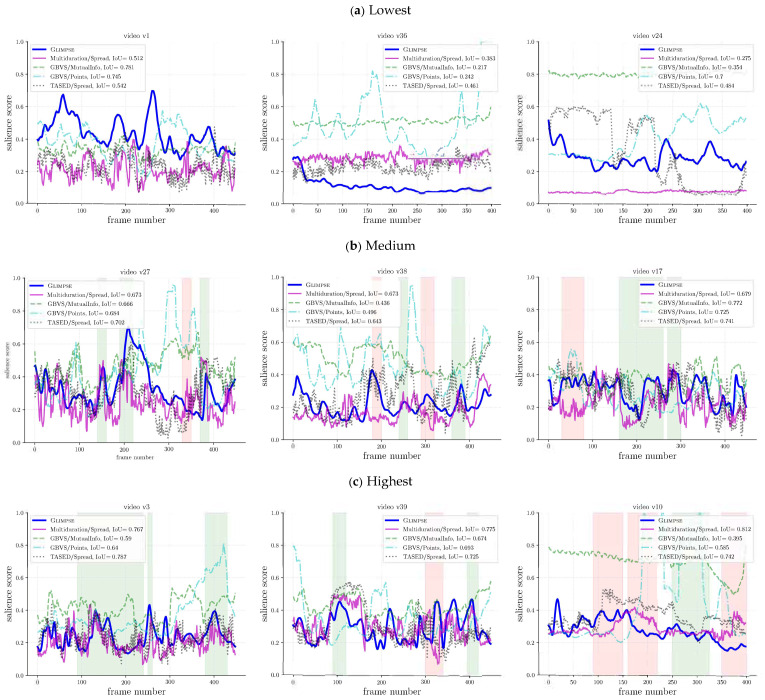
Comparison of s(t) for different methods for the videos where multiduration/Spread gets (**a**) the lowest, (**b**) intermediate, and (**c**) highest IoU. The temporal ranges where multiduration/Spread aligns particular well or poorly with Glimpse are indicated with green and red background, respectively.

We now discuss how the temporal salience scores computed from spatial salience maps relate to the gaze-based scores. We first focus on multiduration/Spread, which was the best performing method according to IoU. For the lowest IoU, the salience scores differed notably; see Figure 9a. For the intermediate IoU values, as can be seen in Figure 9b, although the overall score curves were different in absolute terms, there were interesting matching patterns, some of which are marked with green background regions, but also others where even the reverse patterns were observed, some of which are marked with red background regions. For the highest IoU, the curves may not only be similar in some patterns, but also close in absolute values; see Figure 9c.

Regarding the other computational methods, their behavior was more diverse, but some general patterns could also be identified. For instance, both GBVS/MutualInfo and GBVS/Points tended to overestimate the salience scores, which hardly aligned with Glimpse’s. TASED/Spread performed badly in some videos such as v10, but had good matching patterns in the videos in Figure 9b, which was in agreement with its higher IoU and could be easily noticed around frame t=200. Interestingly, in some cases (e.g., v38), TASED/Spread exhibited a better behavior than multiduration/Spread, which suggested that some of the computational methods may complement one another.

### 5.4. Summary

Computational methods of spatial salience, combined with our scoring algorithms, may be used to estimate temporal salience through some notion of the spatio-temporal consistency of predicted attention. When compared to the reference scores estimated with Glimpse, limited performance was observed. However, interesting matching patterns could be noticed, which suggested that further work is needed for improving the underlying computational model, the scoring strategy, or both. Overall, it can be argued that the best performing computational models are deep-learning based (multiduration and TASED) using the Spread and Points scoring algorithms.

## 6. Discussion, Limitations, and Future Work

The quantification of temporal salience in dynamic scenes such as videos is an overlooked research problem. Arguably, temporal salience may even be more important than spatial salience in these cases [8]. We proposed Glimpse, a novel measure based on the observer-spatio-temporal consistency of gaze points. We showed that Glimpse is conceptually simple and has interesting properties. Crucially, it relies solely on raw gaze data, without analyzing the video contents at all.

Glimpse only has two hyperparameters, the spatial ($\theta_{s}$) and temporal ($\theta_{t}$) scales, which are easily understandable. A potential limitation of our measure is that some domain knowledge may be required to help fine-tune such hyperparameters. For example, in some applications, it may be desirable to smooth the resulting scores with higher $\theta_{t}$ or emphasize the peaks/valleys with lower $\theta_{s}$.

One direction to improve Glimpse would be to include video content analysis. This might help, for example, to automatically and dynamically set the spatial scale $\theta_{s}$ as a function of the size of the relevant object(s) being attended. Furthermore, in our comparison of Glimpse to the temporal salience estimated from spatial salience maps, we used heuristic scoring algorithms, which, being hand-crafted, may miss uncovering relevant visual patterns for more reliable and robust estimates. Therefore, a natural next step is to train a sequential deep neural model using Glimpse’s as the supervisory signal and taking as the input the raw image contents, possibly aided with either precomputed spatial salience maps, or learned end-to-end. This would provide stronger insights into how predictable the gaze-based temporal salience score is from visual-only contents.

Besides the raw gaze data used in this work, the duration of eye fixations could be considered as well, since users typically process information during fixation events [60], so we hypothesize that longer fixations should correlate with higher temporal salience. Comparing scan-paths from multiple observers [61] might be an interesting complementary mechanism for quantifying the temporal attention.

Touching on another promising research line, creating new datasets with ground-truth labels of temporal salience scores is extremely costly, but certainly would facilitate progress in this problem and related topics. Glimpse could be used in this regard, allowing for reliable benchmarking tasks. Another avenue for future work is developing some downstream applications with Glimpse such as video segmentation, compression, summarization, or frame-rate modulation.

Looking forward into the future, we believe Glimpse will contribute to the realization of calm technology [62], where user interaction happens unconsciously. In this context, one could use Glimpse to automatically build annotated datasets of temporal salience with little effort. Considering recent work that has enabled webcams as affordable eye-tracking devices [63] with interesting applications [20], we envision a remote or co-located environment where participants just watch videos at their own pace while their gaze data are collected in the background, aggregated, and processed in a glimpse.

## 7. Conclusions

Glimpse is a novel measure of temporal salience, based on the observer-spatio-temporal consistency of unprocessed eye-tracking data. The measure is conceptually simple and requires no explicit training. Importantly, the estimated salience scores converge quickly with the number of observers, so Glimpse does not need a large number of participants to derive consistent results. Glimpse is computationally efficient, which also lends itself as a suitable method for real-time, on-line computation.

We showed that Glimpse provides consistent estimates of visual attention over time, which could be used in several downstream tasks with video contents. Additionally, we explored scoring algorithms for temporal estimation from computational models of spatial salience. When compared to Glimpse as a reference, they were found to have limited performance.

Ultimately, this paper lays the groundwork for future developments of eye-tracking applications that can make sense of when visual attention is allocated in dynamic scenes. Critically, the distribution of the peaks and valleys of the temporal scores tends to align semantically with salient and human-explainable video events, making our method a sensible approach to produce a consistent reference of temporal salience.

## Figures and Tables

**Table 1:** Details of the SAVAM dataset [25].

**Eye tracker**	SMI iViewXTM Hi-Speed 1250 device at 500 Hz
**Videos**	M=41 FullHD videos (1920×1080 resolution) at 25 fps
	About 13 min of video overall (19,760 frames)
**Participants**	N=58 users (mostly between 18 and 27 years old)

**Table 2:** Glimpse vs. KTS and VisInt, showing 95% confidence intervals.

(a) KTS			(b) VisInt	
precision	recall		$\gamma_{w}=0.8$	$\gamma_{w}=0.2$
[0.130,0.203]	[0.241,0.292]		ρ: [−0.082,0.086]	ρ: [−0.016,0.175]
			τ: [−0.056,0.057]	τ: [−0.006,0.120]

**Table 3:** Asymptotic costs (big O notation) for processing a full HD video having *T* frames.

Method	Cost	Details
KTS	$O(T^{2}m)$	*m*: number of temporal boundaries
VisInt	$O(Tncw^{2}h^{2})$	*n* × *w* × *h*: number × width × height of memory cubes
		*c*: number of image channels
Glimpse	$O(Tn^{2})$	*n*: number of gaze points in the local temporal window

**Table 4:** Runtime costs, reporting the mean±standarddeviation.

Method	Time per Video (s)	Time per Frame (ms)
KTS	11.47±2.80	25.70±5.88
VisInt ($\gamma_{w}=0.2$)	124.88±9.96	280.38±16.37
Glimpse	0.75±0.03	1.69±0.05

## Data Availability

Related data and code are available at https://gitlab.com/vtraver/glimpse (accessed on 29 April 2021).
